# Seed-in-Soil: Pancreatic Cancer Influenced by Tumor Microenvironment

**DOI:** 10.3390/cancers9070093

**Published:** 2017-07-21

**Authors:** Huey-Jen Lin, Jiayuh Lin

**Affiliations:** 1 Department of Medical Laboratory Sciences, University of Delaware, Room 305, Willard Hall Education Building, 16 West Main Street, Newark, DE 19716, USA; 2 Department of Biochemistry and Molecular Biology, Molecular Medicine Graduate Program, University of Maryland School of Medicine and Comprehensive Cancer Center, 108 N. Greene Street, Baltimore, MD 21201, USA; JLin@som.umaryland.edu

**Keywords:** pancreatic ductal adenocarcinoma, tumor microenvironment, carcinoma-associated fibroblasts, signaling pathways, immune-suppression and microRNAs

## Abstract

Pancreatic ductal adenocarcinoma is a fatal malignancy with a five-year survival rate lower than 7%, and most patients dying within six months of diagnosis. The factors that contribute to the aggressiveness of the disease include, but are not limited to: late diagnosis, prompt metastasis to adjacent vital organs, poor response, and resistance to anticancer treatments. This malignancy is uniquely associated with desmoplastic stroma that accounts for 80% of tumor mass. Understanding the biology of stroma can aid the discovery of innovative strategies for eradicating this lethal cancer in the future. This review highlights the critical components in the stroma and how they interact with the cancer cells to convey the devastating tumor progression.

## 1. Introduction

Pancreatic ductal adenocarcinoma (PDAC) accounts for >95% of pancreatic cancer, ranking third highest in cancer-related deaths in the US. Genetic analysis of pancreatic cancer has indicated that multiple mutations accumulate over time, including *KRAS* (about 90%), *p16/ INK4a/CDKN2A* (about 75%), *TP53* (about 65%), and *SMAD4* (about 50%)] [1,2]. Moreover, mutations of *KRAS*, *p16/INK4a/CDKN2A*, and *TP53* result in cells escaping senescence, which allows the tumors to expand [3]. Lack of early symptoms, routine screenings, and effective treatment options, followed by refractory to conventional therapies and the progression of early metastasis to the neighboring vital organs, have led to <5% of patients surviving for more than five years [4]. Only 10–20% of PDAC patients are candidates for surgery at the time of diagnosis, and merely <20% who undergo curative resection are alive after five years [5]. Hence, understanding the biology of PDAC is important in developing improved and effective treatment regimens.

One of the unique characteristics associated with PDAC is that the malignant epithelial cells account for only approximately 20% of the tumor bulk, while the desmoplastic stroma constitute roughly 80% of tumor mass [6]. Hence, it is reasonable to theorize that the aforementioned malignant features may pertain to the unique roles that stroma plays in the aspects of initiating malignancy, escaping immune surveillance, promoting tumor progression and growth, as well as conveying drug resistance and metastasis [7,8,9]. This review highlights the impact of these stromal components on PDAC, with an ultimate goal of eradicating such a deadly cancer. Yet, due to the space limitation, authors regret that some of the outstanding findings cannot be mentioned in this report.

## 2. Cancer-Associated Fibroblasts

PDAC stroma consists of a network that is necessary for supporting tumor growth. The heterogeneous components comprise the carcinoma-associated fibroblasts (CAFs) in an activated state known as pancreatic stellate cells (PSCs), microvasculature, infiltrated immune cells, and the acellular extracellular matrix (ECM) which includes polysaccharides, proteins, cytokines, growth factors, and enzymes [10]. Prior studies illustrated that elevated levels of stroma correlated with poor prognosis, and that ablation of the stromal compartment yielded improved chemotherapy delivery [11,12]. Together, they suggested the tumor-promoting roles that stroma played [11,12]. In supporting this notion, the glycan-binding protein galectin-1 (Gal1) was abundantly expressed in PDAC, and it also plays a stimulating role in the tumor expansion [13]. Genetic ablation of *Gal1* in a mouse model of PDAC (*E1a*-*myc* tumors) weakened tumor progression by impeding proliferation, angiogenesis, hampering desmoplastic reactions, and by favoring immune surveillance, yielding a 20% improvement in survival duration [13]. Furthermore, cancer-associated mesenchymal stem cells were not only isolated from CAFs, and but also secreted granulocyte macrophage colony-stimulating factor (GM-CSF) for augmenting PDAC growth, survival, invasion, and metastasis [14].

CAFs were identified by their expression of another membrane protein known as fibroblast activation protein-α (FAP), which exerted pleiotropic tumor-promoting effects including blocking immune surveillance, adapting PDAC to the host, enhancing tumor vascular density, and augmenting the desmoplastic growth of the microenvironment [15,16]. The conditional depletion of the FAP in CAFs, hence, restored the immune surveillance (that is, anti-tumor) effect not only of the transplanted tumor, but also of an autochthonous model of PDAC [15]. In CAFs, immune suppression by the FAP is facilitated by CXCL12, a chemokine that excludes cytotoxic CD8^+^ T cells by a mechanism depending on the interaction with its receptor CXCR4 [15]. The inhibition of CXCR4 led to diversified tumor-elimination effects by restoring and enabling the rapid intratumoral accumulation of cytotoxic CD8^+^ T cells [15]. Hence, targeting CXCR4 could lead to immune-mediated anti-tumor effects and develop a potential treatment regimen in the near future.

Moreover, CAFs interacted with cancer cells, in part, by releasing chemical messengers packed into miniature double-membraned, cargo-like structures known as CAF-derived exosomes (CDEs) [17]. CDEs contained intact metabolites including amino acids, lipids, and intermediates for citric acid cycle. Together, CDE can reprogram the metabolic machinery following their intake by the cancer cells. Upon CDEs’ ingestion, the mitochondrial oxidative phosphorylation and the normal oxygen-based energy release were dramatically reduced, whereas glycolysis and sugar consumption was enhanced in the cancer cells [17]. Hence, CDEs reprogramed the central carbon metabolism in the cancer cells and further promoted tumor growth, even though the tumors were under nutrient-deprivation conditions [17].

Disappointingly enough, the promising experimental findings stated above have not led to satisfactory clinical applications [18,19,20]. Later studies on stromal biology elicited the discrepancies. For example, Özdemir et al. deleted α-smooth muscle antigen (αSMA) in the myofibroblasts in *PtflaCre/+*; *KrasLSL-G12D/+*; *Tgfbr2flox/flox* (*PKT*) mice, and demonstrated that ablation of myofibroblasts yielded, surprisingly, undifferentiated immune suppression (that is, tumor-promoting) and more invasive PDAC in conjunction with poor prognosis [21]. This finding ignited whether the tumor stroma in PDAC was indeed a double-edged sword, friend, or foe [22]. The mechanisms and functional consequences of the tumor–stroma crosstalk may be more complicated than what was anticipated previously. Single components alone cannot authenticate the biophysical properties or the biochemical complexity around the epithelial cells. Yet, a thorough assessment on crosstalk between multi-factorial parameters in an unbiased manner is required. The sophisticated interactions between the positive and negative growth signals may tip the balance towards tumor suppression or promotion [23,24,25,26].

## 3. Immune Modulation

Tumor-infiltrating immune cells were shown to be vital for tumor progression, metastasis, and chemotherapy resistance [27,28,29]. The mounting of immune-suppressive cells over the course of PDAC included myeloid- derived suppressive cells (MDSCs), T regulatory cells (Tregs), and tumor-associated macrophages (TAMs). Together, they reduced the anti-tumor functionality normally employed by CD8^+^ T cells, and thus resulted in an impairment of tumor recognition and elimination. Initially, tumors secreted GM-CSF for recruiting myeloid progenitor cells to the surrounding stroma, which can be further differentiated into MDSCs [30]. In tumor stroma, MDSCs further blocked the immune surveillance function naturally exerted by the cytotoxic CD8^+^ T cells [26,27]. Recent studies indicated that the interactions between ligands and receptors were important for precluding such an immune scrutiny process. The inhibitory receptors, such as programmed cell death 1 receptor (PD-1, on immune cells), can be masked and blunted by its ligand PD-1L secreted from the tumor cells. Binding of PD-1 to PD-1L abolished the tumor-eradication function that should have been employed by the normal cytotoxic CD8^+^ T cells or by the nature killer cells [31,32]. The outcome of escaping immune surveillance conveyed a permissive tumor microenvironment for cultivating PDAC expansion.

On the other hand, the immune-inhibitory modes ascribed to Tregs involved with the secretion of suppressive cytokines such as interleukin 10 (IL-10), cytotoxic T lymphocyte-associated protein 4 (CTLA-4), and transforming growth factor β (TGFβ) [33]. A subpopulation of CD4^+^ T cells can be influenced by TGFβ stimulation and then be differentiated into interleukin 17 (IL-17)-secreting CD4^+^ T cells (known as Th17) that acquired additional immune-suppressive (that is, tumor-promoting) function [34,35,36]. Interestingly enough, infiltration of Th17 was shown to be aided by oncogenic Kras^G12D^ [37].

Wu et al. elicited that one of the IL-17 cytokine family, IL-17B, played important roles in regulating inflammation, and that delivering neutralizing antibodies reduced tumor burden along with enhanced survival in a mouse xenograft model, manifested by the inhibited tumor proliferation and impeded cancer metastasis [38]. The underlying mechanisms account for this phenomenon were revealed. The binding of IL-17 to its receptor induced the expression of REG3β, which further promoted cell growth and gained refractory to cell death through activation of the gp130-JAK2-STAT3-dependent pathway [39]. Another independent study reported that IL-17B bound to its receptor, IL-17RB, and then induced CCL20/CXCL1/IL-8/TFF1 activation, an event that subsequently rendered noticeable tumor-promoting effects such as the invasion of cancer cells, recruitment of macrophage and endothelial cells at primary sites, as well as resistance of treatments at the distant organs [38]. Taken together, IL-17 plays an intricate role in the pathophysiology of cancer, from tumorigenesis, proliferation, metastasis, to confer both immune and chemotherapy resistance.

Regarding the macrophages, TAMs can be divided into two subtypes according to their developmental states and functionalities: the original state M1 (pro-inflammatory), and the tumor-evolved M2 (immune-suppressive and tumor-promoting). The elevated fraction of M2-polarized TAMs was reported to be correlated with an increased risk of lymph node metastasis, neural invasion, chemoresistance, worsening prognosis, and survival [40,41]. Moreover, M2-polarized TAMs secrete IL-10, which is known to be associated with immune-suppressive and tumor-promoting functionality [42]. The ability of M2-TAMs to enhance tumor invasion and metastasis is not only by preventing tumor cells from being eliminated by CD8^+^ cytotoxic T cells or by natural killer cells, but also by promoting cancer cell proliferation, stimulating extracellular matrix breakdown, and augmenting epithelial-mesenchymal transition (EMT) [43,44], an event that preludes cancer stem cell phenotypes [45]. Under this notion, TAMs were reported to secrete an antimicrobial peptide, hCAP-18/LL-37, which enriched a subpopulation of malignant cells harboring CD133^+^ and displaying cancer stem cell phenotypes [46]. The pivotal transition from the original state, M1, to the tumor-promoting M2 in PDAC, may be one of the major reasons for the poor prognosis of cancer patients. Mounting evidence suggested that M2-polarization was mediated by Reg3β through the activation of the STAT3 pathway in an orthotopic mouse model [47], implicating that abrogating the STAT3 pathway could become a promising therapeutic target.

## 4. Signaling Pathways in PDAC Stroma

### 4.1. Hyaluronan

Hyaluronan (HA) is a glycosaminoglycan with a high capacity of water retention. A high level of HA in PDAC increased intratumoral fluid pressure, created substantial barriers, and impeded the intratumoral penetration of anti-cancer agents [48,49]. The ablation of stromal HA by using PEGylated human recombinant PH20 hyaluronidase (PEGPH20) led to interstitial fluid pressure normalization and re-expansion of collapsed tumor vasculature, followed by improved prognosis in the *KPC* (*KrasLSL.G12D/+*; *p53R172H/+*; *PdxCretg/+*) mice model [49]. In favor of this notion, PEGPH20 plus gemcitabine improved therapeutic outcomes in PDAC patients with high HA tumors [50].

### 4.2. Sonic Hedgehog

Sonic hedgehog (SHh) signaling was recognized as one of the key regulators of tumor epithelia–stromal interaction in PDAC [51]. The SHh ligand, produced by the malignant epithelial cells, signaled to the transmembrane protein Ptch on the stromal cells which subsequently relocated Smo to the cell surface [52,53,54]. This event resulted in the translocation of Gli1 (activator) to the nucleus, followed by a cascade activation of SHh-dependent genes [51,52,53,54].

Olive et al. reported that interrupting SHh signaling using the inhibitor IPI-926 (saridegib) ablated stromal CAFs and led to a transient increase in intratumoral vascular density, followed by an enhanced gemcitabine delivery with an improved cytotoxic outcome in the genetic *KPC* mice model [12]. Despite the aforementioned study providing a promising therapeutic target, another animal model failed to be recapitulated [55]. Similar reports provided paradoxical findings. SHh pathway inhibition suppressed stromal desmoplasia, but accelerated tumor progression of *Kras*-driven mice; whereas activation of SHh signaling caused stromal hyperplasia and reduced epithelial proliferation, leading to a restraint rather than a supporting effect on tumorigenesis [23]. Likewise, Shh-deficient tumors were shown to be more aggressive and they manifested increased vascularity, indicating ablation of stromal fibroblasts led to poor prognosis [24]. The discrepancies among various studies could be due to global and chronic ablation (by genetic knockout) versus the acute blockade of stromal cells (by SHh inhibitors), studying the initiation phase versus established malignant stages, and dosage-dependent as well as off-target effects.

### 4.3. Transforming Growth Factor β (TGFβ)

TGFβ-signaling cascade involved the binding of ligands to their receptors, which furthered the recruitment and phosphorylation of the downstream effectors including the SMAD (mothers against decapentaplegic homologs) family of proteins. Upon activation, SMAD underwent phosphorylation and dimerization, followed by translocation to the nucleus for regulating the expression of downstream TGFβ-dependent genes [56]. The role that TGFβ played in pancreatic cancer was complicated, as it was known to inhibit tumor initiation in the early stages, but favor tumor expansion in later phases [57]. Furthermore, TGFβ affected both the stromal and the neoplastic elements, and this aberrant signaling correlated with poor survival [58].

In favoring the tumor-promoting role, elevated levels of TGFβ have been shown to enhance cell proliferation, suppress immune scrutiny and activate PSCs [59,60,61]. Similarly, Ostapoff et al. demonstrated that introducing a TGFβr2-neutralizing antibody was able to promote a differentiated tumor cell phenotype, and thus inhibit pancreatic cancer metastasis in the orthotopic human tumor xenografts [62]. The underlying mechanisms were involved with targeting the stromal compartment, followed by hampering the activated fibroblasts, collagen deposition, microvessel density, and vascular function [62]. Likewise, the introduction of a TGFβ inactivator (SMAD7) yielded decreased ECM production, reduced fibrosis, and diminished PSCs activation when using a transgenic mouse model [63]. On the other hand, overly activated TGFβ was demonstrated to augment EMT, an event known to initiate metastasis [64], and to sustain cancer stem cell phenotypes [65]. Taken together, the pleiotropic functionality of TGFβ in cancer shall be further investigated, prior to developing a potentially attractive target for treating PDAC.

### 4.4. Abberrant Immune Regulators in Tumor Microenvironment

CD40, a cell surface molecule that belongs to the tumor necrosis factor (TNF) receptor family, was reported to participate in immune regulation and mediate tumor apoptosis [66,67]. CD40 was shown to be one of the key regulators conferring T cell-dependent anti-tumor immunity [66,67]. Under normal physiologic conditions, activation of antigen-presenting cells is aided by CD4^+^ helper cells, which becomes an event that preludes the activation of naïve CD8^+^ T cells into cytotoxic effector cells. Yet, within the PDAC tumor microenvironment, CD40 could override the demand of the CD4^+^ helper cells for activating cytotoxic CD8^+^ T cells. Preclinical studies have evolved the development of CD40-activating antibodies, and they have been tested in clinical trials. One study showed that combination of an agonist CD40 antibody plus gemcitabine resulted in tumor regression in patients who were not eligible for tumor resection [68].

### 4.5. Constitutively Activated Kras Pathway

Kras^G12D^ is required for both the initiation and maintenance processes of pancreatic cancer in mouse models, and was shown to be the most common oncogenic *KRAS* mutation presented in more than 90% of human PDAC, leading to a dominant and constitutively active form of GTPase [69,70]. Such an oncogenic Kras often led to a pathological downstream activation of the phosphoinositide 3-kinase (PI3K) pathway [71]. The factors secreted by Kras for maintaining neoplasm and for promoting stroma appear to include SHh and IL-6 [51,72]. The SHh ligand functioned in a paracrine manner to activate signaling in the stroma and to mediate its maintenance [73]. Molecular studies revealed that SHh induced GLI1 binding to the IL-6 promoter and activated IL-6 expression in fibroblasts in a paracrine fashion [74]. This event further maintained the levels of activated STAT3 in the neighboring cancer cells, acting as a transcription factor required for developing premalignant lesions, maintaining tumor stroma, and advancing neoplastic features [74]. Further molecular studies elicited that Kras^G12D^ activated ERK2 and enhanced the invasion of pancreatic cancer cells via MMP-1 [75]. Oncogenic Kras can also augment the tumor microenvironment by infiltrating immune-suppressing cells that impeded the anti-tumor immune responses. This process subsequently promoted permanent inflammation followed by genetic mutations, which ultimately rendered the aggressiveness of PDAC [27].

Gene expression and metabolic flux analyses further elicited the cancer metabolic role that oncogenic Kras played in orchestrating multiple metabolic changes, including stimulating glucose uptake, differential channeling of glucose intermediates into the hexosamine biosynthesis and pentose phosphate pathways, as well as reprogramming glutamine metabolism [76,77]. By rewiring glucose metabolism while maintaining a low level of reactive oxygen species (ROS), oncogenic Kras limited ROS production and ROS-related apoptosis [76]. Together, biomass synthesis (i.e., proteins, nucleic acids etc.) required for cancer cell proliferation can be boosted [76].

## 5. MicroRNAs

MicroRNAs (miRNAs), the short non-coding RNAs involved in the post-transcriptional suppression of target genes, have been defined as the imperative controllers in tumor proliferations, invasions, and resistance to chemotherapeutic agents. The crosstalk between the malignant epithelia in PDAC and the tumor microenvironment was shown to be interplayed by some miRNAs, such as miR-21 and miR-221 [78]. Ali et al. demonstrated that these miRNAs stimulated the expression of Kras (a target of *miR-221*) as well as enhanced migration and invasion features leading to advanced PDAC [78]. Moreover, miR-155-secreting pancreatic cancer cells furthered the conversion from the normal fibroblasts to CAFs, an event preluded the aggressive malignancy by targeting and downregulating p53-induced nuclear protein 1 (TP53INP1) [79]. Conversely, loss of miR-29 was a common occurrence of activated PSCs, and this phenomenon was correlated with a significant increase in ECM [80]. Hence, correcting and sustaining these miRNAs at their normal levels could likely become promising targets for developing innovative medicine.

Valadi et al. denoted that exosomes can shuttle miRNAs between the donor and the recipient cells that subsequently exerted important biological impacts on the recipient cells [81]. For example, Fabbri et al. revealed that the binding of miR-21 and miR-29a in the exosomes secreted from cancer cells to the Toll-like receptors on the immune cells resulted in an inflammatory response that promoted tumor expansion and metastasis [82]. As exosomes acted as a carrier for miRNAs imperative for conveying a tumor microenvironment conducive to metastasis, they shall not be too far away from constituting potential treatment regimens or becoming biomarkers.

## 6. Cancer Vaccines

Cancer vaccines stimulate the immune system to produce and infiltrate tumor-specific cytotoxic effector T cells by increasing the exposure of tumor-associated antigens to the immune system. The most promising vaccine was GVAX, which was composed of allogeneic PDAC cell lines engineered to secrete GM-CSF [83]. After been administered to the patients with resected or metastatic PDAC, GVAX was able to boost the production of anti-tumor CD8^+^ T cells in peripheral lymphocytes, with an outcome correlated with an improved survival [84]. Another study testing the combination of GVAX and ipilimumab (an antibody blocking CTLA-4) compared to ipilimumab monotherapy showed an appreciative overall survival in metastatic PDAC patients [85]. Likewise, the combination of GVAX with PD-1/PD-L1 blockade together facilitated effector T cell infiltration into pancreatic tumors in a mouse model [32].

Recently, PDAC was recognized to be one of the “nonimmunogenic” malignancies, due to a shortage of tumor-infiltrating effector lymphocytes. Lutz et al. developed an adjuvant clinical trial by combining GVAX with low-dose cyclophosphamide to deplete Tregs. By inducing the infiltration of T cells, patients demonstrated improved survival, enhanced post-vaccination T-cell responses, and increased intratumoral T effector/Treg ratios [86]. Furthermore, Le et al. developed a chimeric GVAX-based vaccine known as GVAXCRS-207 that comprised not only GVAX, but also live-attenuated *Listeria monocytogenes*-expressing mesothelin to stimulate innate and adaptive immunity [87]. Mesothelin was reported to be a common antigen expressed in many human cancers, including PDAC [88]. The delivery of GVAXCRS-207 plus cyclophosphamide to the patients yielded encouraging outcomes with extended survival and minimal cytoxicity [87]. Taken together, GVAX appeared to be very specific, and vaccine therapies were relatively tolerated. Future innovative treatment regimens could adapt the synergistic effect from GVAX along with other target agents. 

Another DNA-based vaccine comprised of Mucin 1 plus variable number tandem repeat (MUC1-VNTR_6_, each repeat of VNTR encodes 20 amino acids GVTSAPDTRPAPGSTAPPAH) was recently developed, and was transfected to immature dendritic cells [89]. Upon intake of the plasmid construct pVAX1-MUC1-VNTR_6_, dendritic cells not only yielded elevated immunogenicity, but their neighboring co-cultured T-cells also gained evident cytotoxicity, which was manifested by their growth inhibitory effect on PDAC in both laboratory cultivation experiments and tumor-bearing animal studies [89].

## 7. Concluding Remarks and Future Treatment Regimens

**Figure 1:**
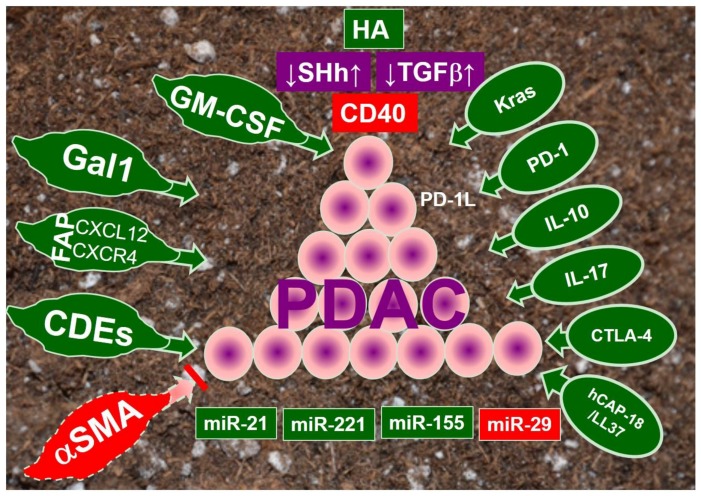
The pleiotropic influence of the tumor microenvironment on pancreatic ductal adenocarcinoma (PDAC). The progression of (PDAC) (pyramid spheres in the center) is controlled by various molecules and signaling pathways in the tumor microenvironment. While molecules associated with carcinoma-associated fibroblasts or pancreatic stellate cells were displayed in spindles at the left side of the figure, others involved with immune cells are presented in ova at the right side of figure. Moreover, the signaling pathways are presented in rectangles above PDAC, while the microRNAs (miR) are underneath. Tumor-promoting factors are shaded in green, while inhibiting factors are in red. Yet, ones shaded in purple are pleotropic, with both promoting and inhibiting effects, depending on the stage of tumors as well as the research studies. The abbreviations used are CDEs (CAFs-derived exosomes); CTLA-4 (cytotoxic T lymphocyte-associated protein 4); FAPa (fibroblast activation protein-a); Gal1 (glycan-binding protein galectin-1); GM-CSF (granulocyte macrophage colony-stimulating factor); HA (hyaluronan); IL-10 (interleukin 10); IL-17 (interleukin 17); Kras (constitutively oncogenic Kras); miR (microRNA); PD-1 (programmed cell death 1 receptor); PD-1L (ligand for PD-1); and SHh (Sonic hedgehog).

In summary, Figure 1 depicted the progression of PDAC can be orchestrated by various tumor microenvironmental elements, and some of them have been utilized for developing targeted therapies (see Table 1). Future improved treatments for PDAC could include combination regimens aiming to normalize desmoplastic reaction, inhibit tumorigenic-signaling pathways, reprogram immune suppression (known as immunotherapy), correct aberrant miRNAs, and implement cancer vaccines.

## Figures and Tables

**Table 1:** Summary of selected agents targeting microenvironmental factors in pancreatic cancer.

Target	Clinical Studies	Reference
Hyaluronan and chemotherapy agent	Phase 1b PEGPH20 plus Gemcitabine	[50]
Transforming Growth Factor β	Orthotopic human tumor xenografts TGFβr2 neutralizing antibody (2G8)	[62]
Cancer vaccine	GVAX. Phase 2	[83,84]
Cancer vaccine and CTLA-4	GVAX and ipilimumab	[85]
Cancer vaccine and Treg	GVAX and cyclophosphamide	[86]
Cancer vaccine, Treg, and mesothelin	Cyclophosphamide/GVAXCRS-207	[87]
